# ArduiTaM: accurate and inexpensive NMR auto tune and match system

**DOI:** 10.5194/mr-1-105-2020

**Published:** 2020-06-29

**Authors:** Mazin Jouda, Saraí M. Torres Delgado, Mehrdad Alinaghian Jouzdani, Dario Mager, Jan G. Korvink

**Affiliations:** 1 Karlsruhe Institute of Technology (KIT), Institute of Microstructure Technology, Karlsruhe 76131, Germany

## Abstract

We introduce a low-complexity, low-cost, yet sufficiently accurate automatic tune and match system for NMR and MRI applications. The ArduiTaM builds upon an Arduino Uno embedded system that drives a commercial frequency synthesiser chip to perform a frequency sweep around the Larmor frequency. The generated low-power signal is fed to the NMR coil, after which the reflected waves are detected using a directional coupler and amplified. The signal shape is then extracted by means of an envelope detector and passed on to the Arduino, which performs a dip search while continuously generating actuator control patterns to adjust the tune and match capacitors. The process stops once the signal dip reaches the Larmor frequency. The ArduiTaM works readily with any spectrometer frequency in the range from 1 to 23 T. The speed of the ArduiTaM is mainly limited by the clock of the Arduino and the capacitor actuation mechanism. The Arduino can easily be replaced by a higher-speed microcontroller, and varactors can replace stepper-motor controlled variable capacitors. The ArduiTaM is made available in open source, and so is easily duplicated.

## 1 Introduction

Nuclear magnetic resonance spectrometers for imaging and spectroscopy are predominantly operated with inductor–capacitor (LC) resonators that pick up the radio frequency signal due to the evolving spin magnetisation in a sample or patient. Because the detected NMR signals are naturally very weak, it is of utmost importance that high-quality-factor components are used along the analogue radio frequency signal path, so as to minimise signal losses.

Proper tuning and matching (T&M) of the NMR probe is also important, and when done properly as will be demonstrated in the following section, this significantly enhances the receiver's sensitivity, thereby reducing the signal averaging times that would otherwise be required to obtain sufficient signal-to-noise ratio (SNR).

As detailed in [Bibr bib1.bibx1], sample loading can significantly alter the electrical properties of the NMR coil and thereby disturb its tuning-and-matching condition (see Fig. S1 in the Supplement). Therefore, the NMR coil is usually re-tuned and re-matched after loading a new sample. In many NMR/MRI systems, T&M is done manually by mechanically adjusting two trimmer capacitors. Although this hands-on process has been acceptable for many years, the need for automatic procedures has become important with the trend towards high-throughput experiments, for example so that samples can be automatically loaded into the magnet and analysed without delay.

The major commercial system providers recently started offering add-ons to equip their probes with automatic tuning and matching. These systems contain actuation units that drive the mechanical trimmer capacitors of the probe and are, in turn, controlled by the spectrometer software, which uses a so-called wobble routine to obtain the feedback signal. The offered solutions are costly and therefore only practical for probes with a limited number of channels. A further drawback is that the commercial systems are designed to drive mechanical trimmers, and therefore are not adaptable to tuning and matching customised probes that for example involve digital capacitors or varactors. Moreover, these commercial systems are further limited by the capabilities of the RF channels of the spectrometer, and thus lack the generality needed by the experimentalist. So, for instance, an automatic T&M system installed in an NMR spectrometer with a ${}^{1}$ H narrow-band channel cannot be used to T&M a ${}^{13}C$ coil, or vice versa.

To overcome these limitations, we considered the availability of off-the-shelf analogue and digital electronic components needed for such a system. In this paper, we report our findings and present a compact low-cost accurate Arduino-based automatic tuning and matching system, abbreviated as ArduiTaM. Unlike commercial solutions and previously published reports [Bibr bib1.bibx2] that use either the MR spectrometer or a commercial network analyser for frequency generation and signal processing, our system employs a single chip frequency source covering a useful range of 35 to 4.4 GHz, as well as discrete signal processing electronics, rendering it a completely standalone system capable of tuning and matching almost any relevant NMR/MRI probe channel. The ArduiTaM is compatible with most NMR spectrometers, can readily be interfaced via two TTL lines (almost all NMR/MRI systems provide general purpose TTL inputs and outputs that can be arbitrarily programmed by the user), and requires neither software add-ons (like Koczor's work, [Bibr bib1.bibx4]) nor hardware alterations. The spectrometer probe can thus be brought back to its original state by simply detaching the unit.

The ArduiTaM accomplishes T&M using the same principle that one would apply manually, namely, it monitors the signal reflected from the probe ( $S_{11}$ ) and then varies the tune and match capacitors until the minimum signal reflection amplitude occurs at the Larmor frequency. This gives it the advantage of being completely independent of the probe's topology, thereby eliminating constraints on the probe, such as the orthogonality of tuning and matching considered in Hwang's paper [Bibr bib1.bibx2]. Furthermore, the ArduiTaM can, apart from NMR/MRI experiments, be a handy tool in any RF laboratory, useful to characterise coils, coil arrays, antennas, and impedance matching networks.

## 2 Why tune and match?

In almost all commercial NMR systems the spectrometer electronics racks are placed adjacent to the magnet due to their relatively large dimensions. This necessitates the use of shielded coaxial cables to guide the NMR signals from the coil to the spectrometer. The coaxial cables usually have a characteristic impedance, $Z_{0}$ , of 50 Ω , and therefore all RF components of the NMR spectrometer (such as the power amplifier of the transmitter and the low-noise amplifier of the receiver) are designed accordingly to ensure impedance matching and consequently maximise power transfer. The NMR coil is no exception, and the use of a 50 Ω coaxial cable to connect the coil to the spectrometer implicitly implies that it also must be matched to $Z_{0}$ to guarantee an efficient excitation field, $B_{1}$ , in the excitation phase, and an efficient signal acquisition in the reception phase of the NMR experiment. The impedance matching of the NMR coil also minimises the signal reflection coefficient Γ ,

1 $$\Gamma =\frac{Z_{\mathrm{coil}}-Z_{0}}{Z_{\mathrm{coil}}+Z_{0}},$$

and thus prevents the formation of standing waves in the cables [Bibr bib1.bibx5].

**Figure 1 Ch1.F1:**
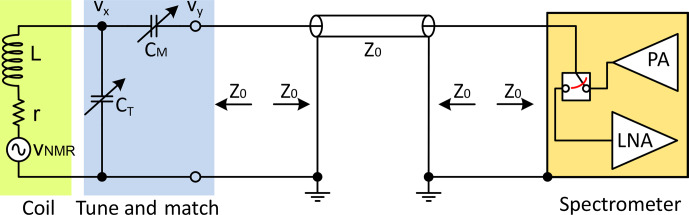
Example of a widely used parallel-tune/series-match topology. The L -matching circuit converts the complex impedance of the coil to a purely resistive impedance equal to the characteristic impedance of the coaxial cable, $Z_{0}$ .

Matching the coil impedance is commonly realised using high-quality, almost lossless, capacitors, as illustrated in Fig. [Fig Ch1.F1]. As such, the matching process also implies that the coil will be part of a resonant circuit, hence the term “tuning”. The process of tuning and matching the coil in Fig. [Fig Ch1.F1] can be performed as follows: first, $C_{T}$ is varied to make the coil resonate at a frequency, $\omega_{r}$ , slightly above the Larmor frequency, $\omega_{l}$ , such that the real impedance of the parallel resonator is 50 Ω at $\omega_{l}$ . Then, the residual imaginary impedance, which in this case is inductive, can be eliminated by varying $C_{M}$ . In effect, the extra capacitance is the complex conjugate of the residual inductance. This process results in the coaxial cable experiencing a purely resistive 50 Ω load impedance at $\omega_{l}$ . Due to resonance, the tuning and matching network acts, in what can be considered one of its major advantages, as a passive noiseless preamplifier, thus $\left|v_{y}|=G_{\mathrm{TM}}\cdot |v_{\mathrm{NMR}}\right|$ , with the tune and match voltage gain ${\text{G}}_{\mathrm{TM}}=0.5\sqrt{Z_{0}/r}$ . According to Friis' formula,

2 $$F_{\mathrm{Total}}=1+\frac{F_{\mathrm{receiver}}-1}{G_{\mathrm{TM}}};$$

this particular feature can significantly lower the noise factor F (noise figure NF=10log⁡F ) of the NMR receiver and thus enhance its sensitivity (see Fig. S2 in the Supplement).

**Figure 2 Ch1.F2:**
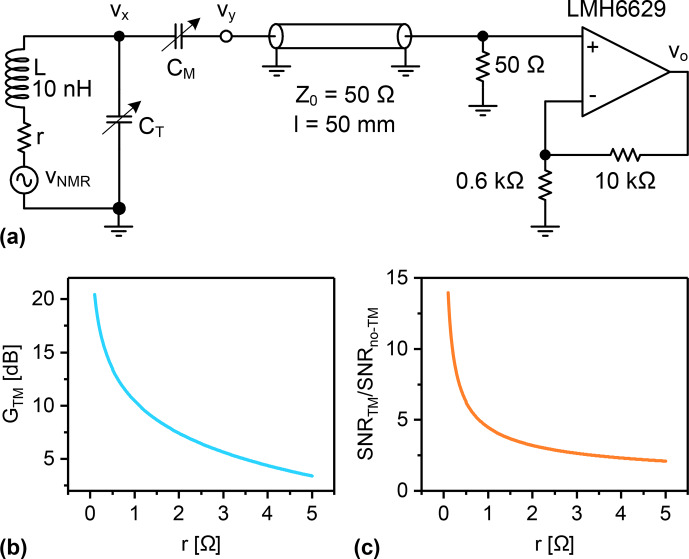
The effect of T&M the NMR coil on the overall signal-to-noise ratio after the receiver. **(a)** Typical NMR receiver circuit including the coil, T&M capacitors, coaxial cable, and low-noise amplifier. The cable length was set to be short enough so that its attenuation is negligible. **(b)** The passive amplification, $G_{\mathrm{TM}}$ , as a function of the coil's resistance. **(c)** The overall SNR enhancement as a function of the coil's resistance. All simulations were performed using the Advanced Design System (ADS) software.

To obtain an impression of how significant this can be, consider the circuit in Fig. [Fig Ch1.F2]a as an example of a typical MR receive channel. The circuit uses the LMH6629 low-noise amplifier from Texas Instruments^®^ in a non-inverting topology. The operation frequency of the circuit is arbitrarily set to 500 MHz and the T&M capacitors are assumed to be noiseless. The circuit was simulated using the Advanced Design System software (ADS) to explore the effect that T&M the NMR coil will have on the overall signal-to-noise ratio. Figure [Fig Ch1.F2]b shows the voltage amplification, $G_{\mathrm{TM}}=|v_{y}/v_{\mathrm{NMR}}|$ , due to T&M, while Fig. [Fig Ch1.F2]c demonstrates the ratio of the output SNR with T&M, to that without T&M, for different values of the coil's AC resistance. The latter figure clearly shows how T&M can significantly enhance the SNR. The enhancement falls off as the coil's noise increases. For a coil with a relatively high AC resistance of, say 5 Ω , T&M still results in an SNR enhancement by a factor of more than 2. Thus improper T&M results not only in an inefficient power transfer during transmission, but also in an increase in the NF during reception, leading to a severe loss of sensitivity.

**Figure 3 Ch1.F3:**
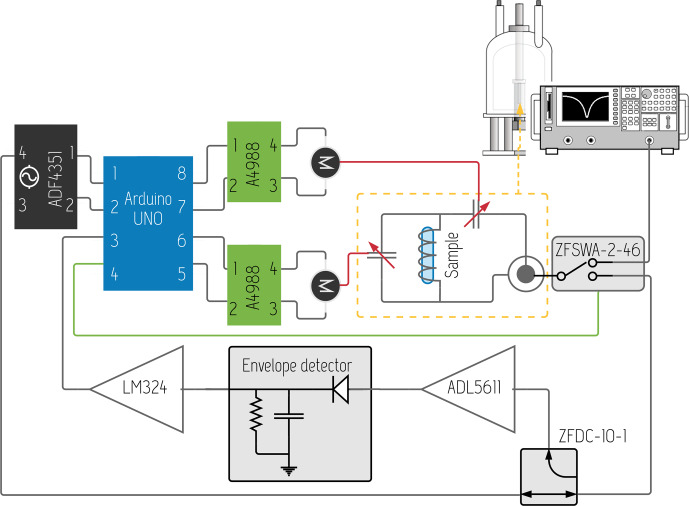
Block diagram of the ArduiTaM, detailing its component interconnections, as well as how the system can be inserted into the signal path of a spectrometer.

**Figure 4 Ch1.F4:**
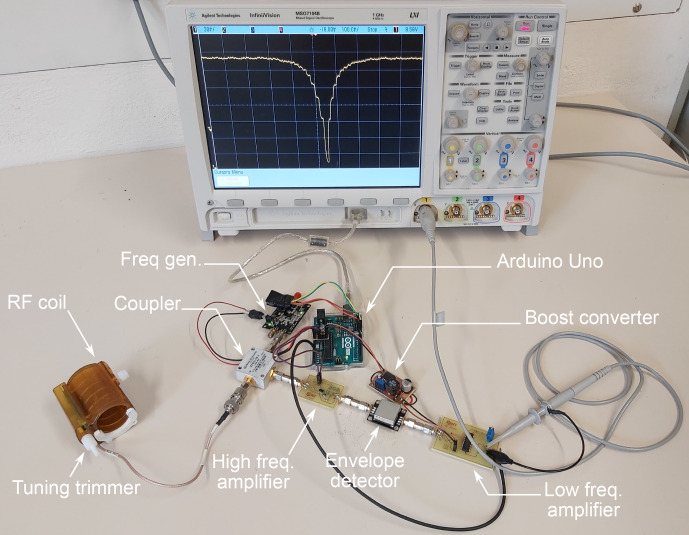
The experimental setup of the ArduiTaM showing the acquired $S_{11}$ signal of the coil on the oscilloscope.

## 3 ArduiTaM implementation

The ArduiTaM requires hardware and software co-design, which is detailed in this section.

### 3.1 Circuit design

Figure [Fig Ch1.F3] depicts the block diagram of the ArduiTaM circuit, for the case of T&M capacitors that are rotated using stepper motors. This figure also describes how the ArduiTaM is easily inserted into the signal path of a spectrometer, underlining its integrability. It requires minimal connections, namely, a TTL trigger signal from the spectrometer to the Arduino, and a TTL acknowledgement signal from the Arduino back to the spectrometer, to facilitate automation. After loading a new sample, the spectrometer can thus trigger the ArduiTaM to readjust the T&M of the probe. Once the ArduiTaM is done, it will send an acknowledgement signal back to the spectrometer so as to start the NMR experiment.

The ArduiTaM circuit consists of an Arduino Uno as the master controller, carrying out the frequency sweep control, signal acquisition, signal processing, optimum T&M condition search, and adjustment of the variable capacitors. The Arduino furthermore allows the user to set the channel Larmor frequency, as well as the frequency sweep range for tuning and matching. The ArduiTaM uses an ADF4351 high-quality ultra-wideband frequency synthesiser from Analog Devices^®^ to generate the required frequency sweeps. The synthesiser is controlled using an SPI protocol and covers a frequency range from 35 MHz to 4.4 GHz, making the ArduiTaM compatible with the frequencies of almost any commercial NMR spectrometer available today. So, for example, the ArduiTaM can T&M any of the following: a 300 MHz ${}^{1}$ H probe in a 7 T magnet, a 125 MHz ${}^{13}C$ probe in a 11.7 T magnet, a 600 MHz ${}^{1}H$ probe in a 14.1 T magnet, and so on.

The output of the synthesiser is transferred to the NMR probe through a low-loss directional coupler (ZFDC-10-1 from Mini-Circuits^®^) and then through a high-quality (low insertion loss and high isolation) RF switch (ZFSWA-2-46 from Mini-Circuits^®^). The latter is used to toggle between the “T&M mode” in which the NMR probe is connected to the ArduiTaM and the “NMR mode” in which the probe is connected to the NMR spectrometer, and is the only additional lossy element that is active during NMR signal acquisition (also refer to Fig. [Fig Ch1.F9]).

The reflected signal from the probe is taken from the coupler and amplified using an ADL5611 from Analog Devices^®^ (all circuit schematics are available in the Supplement). After that, the envelope of the reflected signal is extracted using a high-sensitivity ( -40 dBm) pin-diode-based envelope detector. The extracted envelope represents the probe's signal reflection, or $S_{11}$ curve, and is further amplified using an LM324 with an explicit low-pass filter of 200 Hz bandwidth to eliminate high-frequency residuals. The low-frequency $S_{11}$ curve is acquired by the Arduino through one of its analogue inputs, and the auto T&M algorithm is executed based on the acquired data. Figure [Fig Ch1.F4] shows the interconnected discrete components of the ArduiTaM in which an oscilloscope was used to visualise the acquired $S_{11}$ curve of the coil, verifying performance.

**Figure 5 Ch1.F5:**
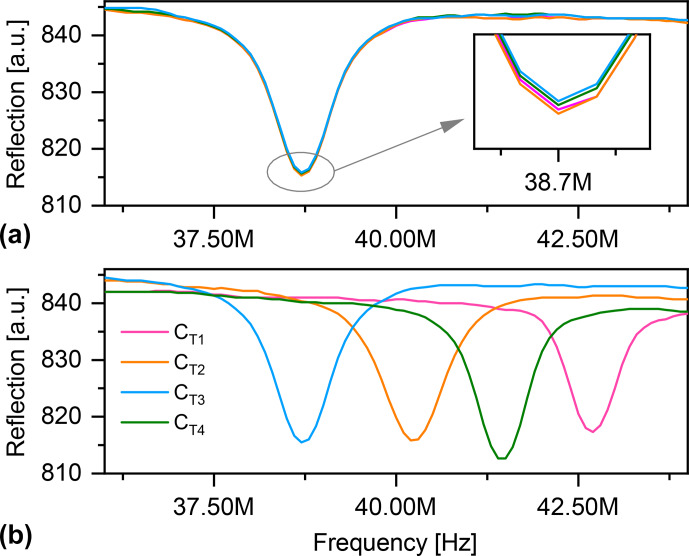
**(a)** Four consecutive acquisitions of $S_{11}$ , by the ArduiTaM, of a coil with a fixed $C_{T}$ value. The overlaid curves were acquired via one of the analogue inputs of the Arduino. The x axis covers the frequency range of the sweep. The y axis represents the magnitude of the reflected signal. The insert zooms in onto the minimum of the curves. **(b)** $S_{11}$ of the same coil with four arbitrarily different $C_{T}$ values.

Figure [Fig Ch1.F5] displays four acquisitions of the $S_{11}$ curves recorded by the Arduino for two cases: when the tuning capacitor, $C_{T}$ , of the coil is (a) fixed, and (b) arbitrarily changed. The results demonstrate the robustness and reliability of the circuit.

### 3.2 Tuning algorithm

Before and after the T&M protocol runs, a trigger signal is sent to and from the Arduino microcontroller to toggle between two modes of operation: T&M and NMR measurement by the host system. Once the start trigger signal is received, the algorithm initialises all variables and resets the position of the capacitors, $C_{t}$ and $C_{m}$ , to their minimum value using a so-called “homing routine”. The routine causes the stepper motors to turn anticlockwise until they reach a lower angle limit, corresponding to the lowest value of the trimmer capacitors. Afterwards, the user is asked to enter the desired Larmor frequency, $f_{0}$ , for which the tuning and matching conditions will be sought.

The algorithm sweeps the tuning capacitances against fixed values for the matching capacitances, capturing the $S_{11}$ values for each combination. $S_{11}$ values are read from an analogue input of the microcontroller at which the already amplified and extracted envelope signal is input. To reduce the algorithm's running time, the first sweep is done at a low resolution, with a sweep step size of $S_{\mathrm{sw}}=64\cdot S_{m}$ , where $S_{m}$ represents a single step of the stepper motor, which is equivalent to the inverse of the steps per turn, referred to here as “spt”, that a stepper motor can provide. Our prototype uses motors with spt=200 , resulting in $S_{m}=1.8$ ${}^{\circ }$ . Once the first sweep is complete, the capacitor pair $\left\{C_{T},C_{m}\right\}$ with the lowest $S_{11}$ value ( $S_{11\;\min}$ ) is found and a new searching window is defined. The chosen search intervals are symmetric around $S_{11\;\min}$ with a span of $S_{\mathrm{sw}}$ in each direction, i.e. $S_{11\;\min}\pm S_{\mathrm{sw}}$ , for which the corresponding tuning and matching capacitor values are already known. The same cycle is run four consecutive times, increasing the resolution by 4 with respect to the previous cycle until the maximum resolution of rotational increment is reached, that is, $S_{\mathrm{sw}}=S_{\mathrm{sw}}/4$ until $S_{\mathrm{sw}}=S_{m}$ (see Fig. [Fig Ch1.F6]). Ultimately, the algorithm's resolution is limited by $S_{m}$ , which can be easily improved with the use of stepper motors with a higher spt value, or through the use of a step down gear box. After the last cycle, the motors are positioned at the angles for which tuning and matching capacitances corresponded to the lowest probe reflection. Finally, the microcontroller activates the RF switch and sends a trigger signal to the NMR spectrometer to switch to NMR measurement mode. The current implementation requires the user to do one-time single-point calibration of the tuning and matching capacitors when attaching the ArduiTaM for the first time. This can be easily done by manually rotating the trimmers to their lower values, which are recognised by the ArduiTaM as the zero positions, and by setting the allowed number of turns before upper values are reached. As long as it is not turned off, the ArduiTaM can keep track of stepper motors' positions and ensure that neither limit is exceeded. A simple modification of the code, such that current positions of the trimmers are written in the EEPROM of the Arduino, would allow the ArduiTaM to keep track of the motors' positions even after being completely powered off.

**Figure 6 Ch1.F6:**
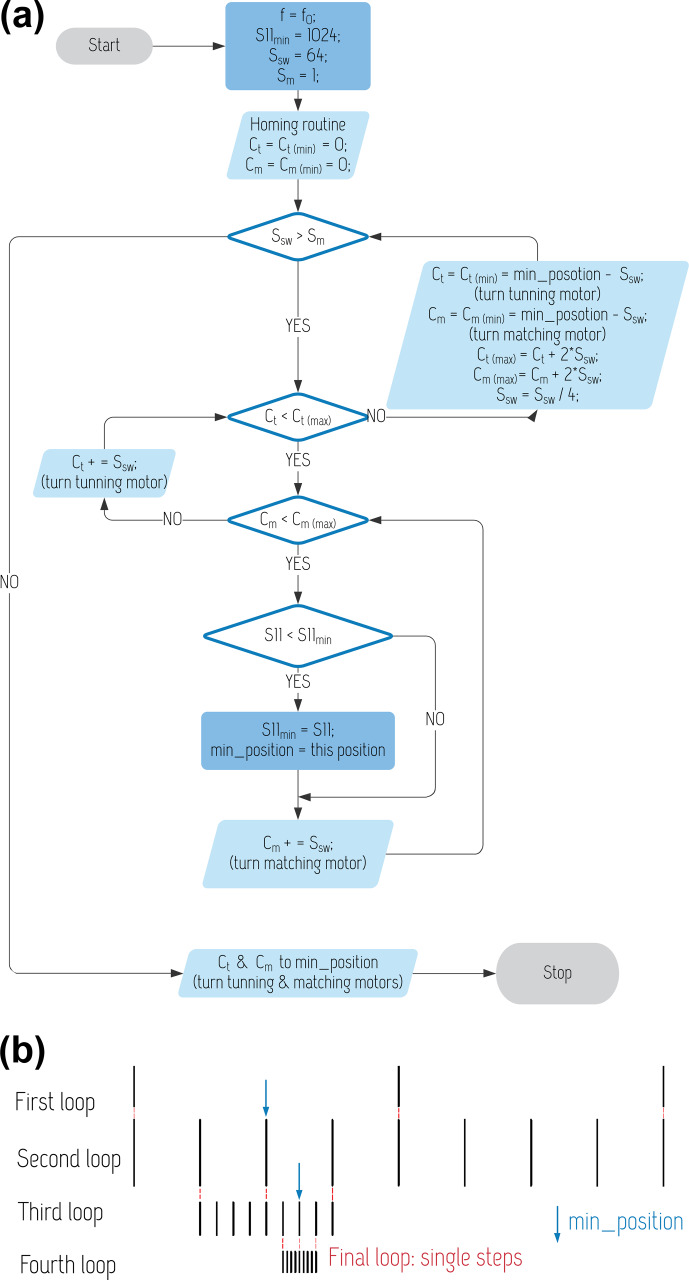
**(a)** T&M flowchart. **(b)** Schematic showing the interval subdivision method to zoom in on the $S_{\mathrm{aa}}$ minimum.

**Figure 7 Ch1.F7:**
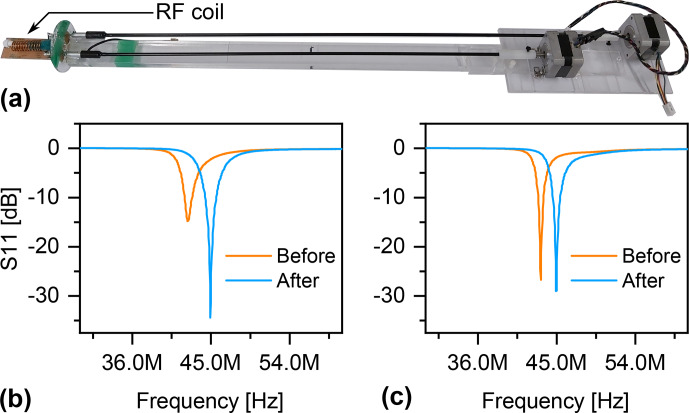
**(a)** Homemade RF coil with a mechanical setup for automatic T&M. The coil operates at a Larmor frequency of 44.93 MHz. **(b)** Result of applying the ArduiTaM to T&M the coil starting from an arbitrary condition. **(c)** Result of applying the ArduiTaM to adjust the tuning capacitor only.

**Figure 8 Ch1.F8:**
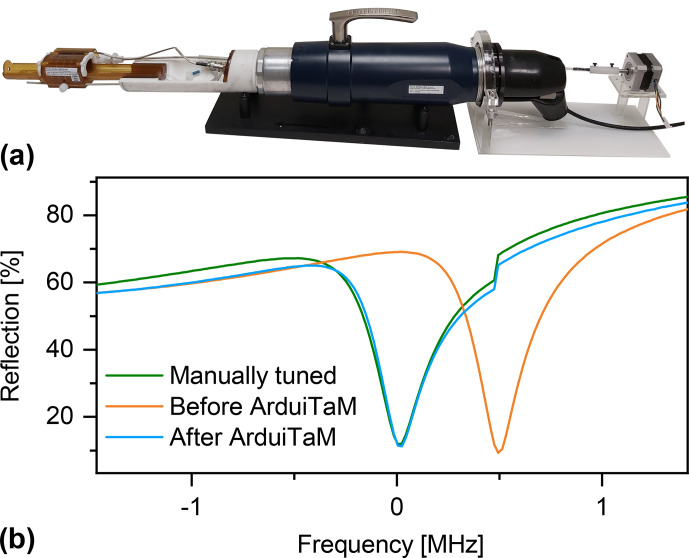
**(a)** Integrating the ArduiTaM with a commercial MRI probe (Bruker ICON). **(b)** “Wobble” curves showing the coil's reflection when it is manually tuned, and before and after the automatic tuning using the ArduiTaM.

## 4 Results

The ArduiTaM was first tested on a homemade RF coil. The solenoid was designed to operate in a 1.055 T preclinical MRI magnet at a Larmor frequency of 44.93 MHz. We used two sapphire dielectric trimmers, each with 28-turn threads, covering a range of 0.5 to 10 pF, to tune and match the coil according to the circuit topology depicted in Fig. [Fig Ch1.F2]. Two stepper motors were used to mechanically drive the trimmers; see Fig. [Fig Ch1.F7]a. In order to avoid potential mutual interactions, the motors were placed outside the magnet where the average stray field is around 500 µT . In this setup we explored two scenarios.

1. The two trimmers were arbitrarily set, and the ArduiTaM was applied. Covering a tuning and matching range of six turns for each trimmer, and using four loops of resolution (corresponding to a final step of approximately 32 fF), the ArduiTaM could precisely T&M the coil in 2 min and 24 s. Figure [Fig Ch1.F7]b shows the $S_{11}$ curve of the coil before and after applying the ArduiTaM.
2. The matching capacitor of the coil was adjusted to achieve an optimum matching at the Larmor frequency, whereas the tuning capacitor was arbitrarily set. Covering a wider sweep range of nine turns with a finer resolution through six loops, the ArduiTaM was capable of precisely tuning the coil, Fig. [Fig Ch1.F7]c, in 22 s.

The speed of rotation of the stepper motors was kept conservatively low to protect the capacitors from mechanical damage, which affected the time of the measurement. The $S_{11}$ measurements in Fig. [Fig Ch1.F7] were obtained using a Keysight E5071C network analyser.

In order to highlight its practicality, we applied the ArduiTaM to a commercial probe (Bruker ICON) as shown in Fig. [Fig Ch1.F8]a. The probe is designed such that its matching condition is always met over the entire range of targeted samples. Therefore, it exhibits a tuning capacitor only. We used an RF switch (ZFSWA-2-46), controlled by the TTL output of the MRI scanner, to toggle between the T&M mode where the ArduiTaM runs and the MRI mode in which the scanner's electronics are routed to the coil. Figure [Fig Ch1.F8]b plots the percentage of reflection from the coil, recorded using the standard “wobble” routine of the scanner, for three cases; first, when the coil was manually tuned, second, when the coil was arbitrarily de-tuned, and third, when the coil was automatically re-tuned using the ArduiTaM. The tuning range, the number of resolution loops, and thus the tuning time (22 s) were similar to the values used in Fig. [Fig Ch1.F7]c.

**Figure 9 Ch1.F9:**
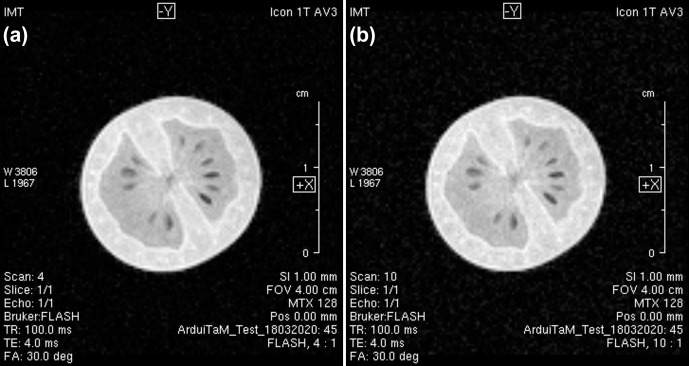
Integrating the ArduiTaM with a commercial MRI scanner shows only a negligible effect on its performance. **(a)** MR image of a cherry tomato before applying the ArduiTaM. The measured SNR is 49. **(b)** Repeating the same experiment after applying the ArduiTaM. The measured SNR here is 43, reflecting a loss of approximately 12 %, due to the insertion loss of the RF switch (ZFSWA-2-46 from Mini-Circuits^®^).

In order to assess the effect of the ArduiTaM on the performance of a commercial system, we conducted an imaging experiment of a cherry tomato with and without applying the ArduiTaM; see Fig. [Fig Ch1.F9]. For both experiments, we utilised a standard gradient-echo sequence with a 1 mm slice thickness, 312 µm in-plane resolution, 30 ${}^{\circ }$ flip angle (FA), 4 ms echo time (TE), 100 ms repetition time (TR), and averaging eight times. Moreover, to ensure that both experiments have exactly the same initial settings, we ran the automatic adjustment routines before each experiment. These include a “drift adjustment” to correct the frequency drift caused by the field drift, a “power adjustment” to calculate the correct power that corresponds to the desired flip angle, and a “receiver gain adjustment” to calculate the receiver gain that ensures optimum digitisation of the signals. The measured linear-scale SNR before applying the ArduiTaM, Fig. [Fig Ch1.F9]a, is 49, while the SNR after inserting the ArduiTaM, Fig. [Fig Ch1.F9]b, is 43 corresponding to a 12 % (1.13 dB) loss in the signal-to-noise ratio. The slight loss in SNR is due to the insertion loss of the RF switch (0.8 dB) needed by the ArduiTaM to route the RF signals.

## 5 Conclusions

The ArduiTaM is a fully functional automatic tuning and matching system for use in NMR or MRI systems. Despite its simplicity, the ArduiTaM exhibits high precision, reliability, and, most importantly, a negligible influence on NMR signal fidelity. By utilising an ADF4351 frequency synthesiser, the ArduiTaM is completely independent of the spectrometer and conveniently covers a frequency range of 35 MHz to 4.4 GHz, making it compatible with most commercial NMR or MRI frequencies. Moreover, its very low cost (around EUR 100 for the chip components at the time of writing) makes it particularly attractive for use with multi-resonant probes and MRI phased arrays. Away from MR, other potential applications, such as antennas, resonators, and impedance matching networks, may stand to benefit as well.

The ArduiTaM was successfully tested on a homemade coil prototype that uses stepper-motor-driven trimmer capacitors for tuning and matching. Trimmers were purposely employed to demonstrate the ArduiTaM's ease of integrability with commercial systems, where such capacitors are predominantly used. Unfortunately, the use of mechanical trimmers imposes a limit on the speed of T&M. However, trimmers can easily be replaced with varactors or digitally tuned capacitors (DTCs), for much faster tuning and matching. The speed of T&M can be further optimised depending on the experiment. So, for example, if the maximum frequency shift happens to be known for the samples under test, then the sweep ranges can be minimised so that the ArduiTaM can make more rapid adjustments.

## Supplement

10.5194/mr-1-105-2020-supplement The supplement related to this article is available online at: https://doi.org/10.5194/mr-1-105-2020-supplement.

## Data Availability

All circuit schematics are provided in the Supplement. The Arduino codes are available at https://doi.org/10.5281/zenodo.3908231 (Jouda, 2020).
