# Supplementary material for: ArduiTaM: accurate and inexpensive NMR auto tune and match system
Source: Magn Reson (Gott). 2020 Jun 29;1(1):105–13. doi: 10.5194/mr-1-105-2020 (PMC10500709; doi:10.5194/mr-1-105-2020)
Supplement: The supplement related to this article is available online at: https://doi.org/10.5194/mr-1-105-2020-supplement. [file mr-1-105-supplement.pdf]

Supplement of Magn. Reson., 1, 105–113, 2020
https://doi.org/10.5194/mr-1-105-2020-supplement
© Author(s) 2020. This work is distributed under
the Creative Commons Attribution 4.0 License.

Supplement of

ArduiTaM: accurate and inexpensive NMR auto tune and match system

Mazin Jouda et al.

Correspondence to: Jan G. Korvink (korvink@kit.edu)

The copyright of individual parts of the supplement might differ from the CC BY 4.0 License.

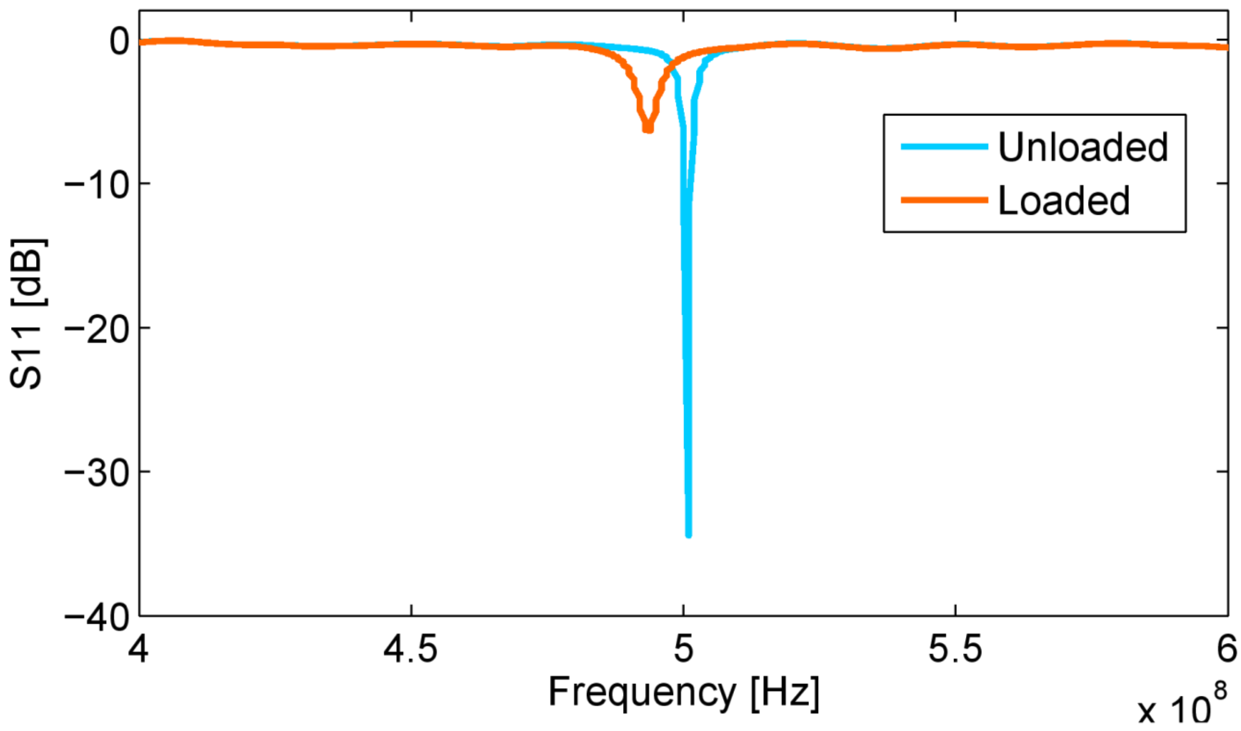

Fig. S1: Effect of sample loading on the tuning and matching conditions. The coil is a commercial 5mm saddle coil designed to operate in a 11.7 T Bruker magnet at 500 MHz. Loading the sample (0.5 M NaPO3, 0.5 M Phosphoric Buffer Solution (PBS), 50 mM TSP, 0.5 M Sucrose) disturbs the tuning and matching condition largely (6 MHz frequency shift, 28 dB change in S11).

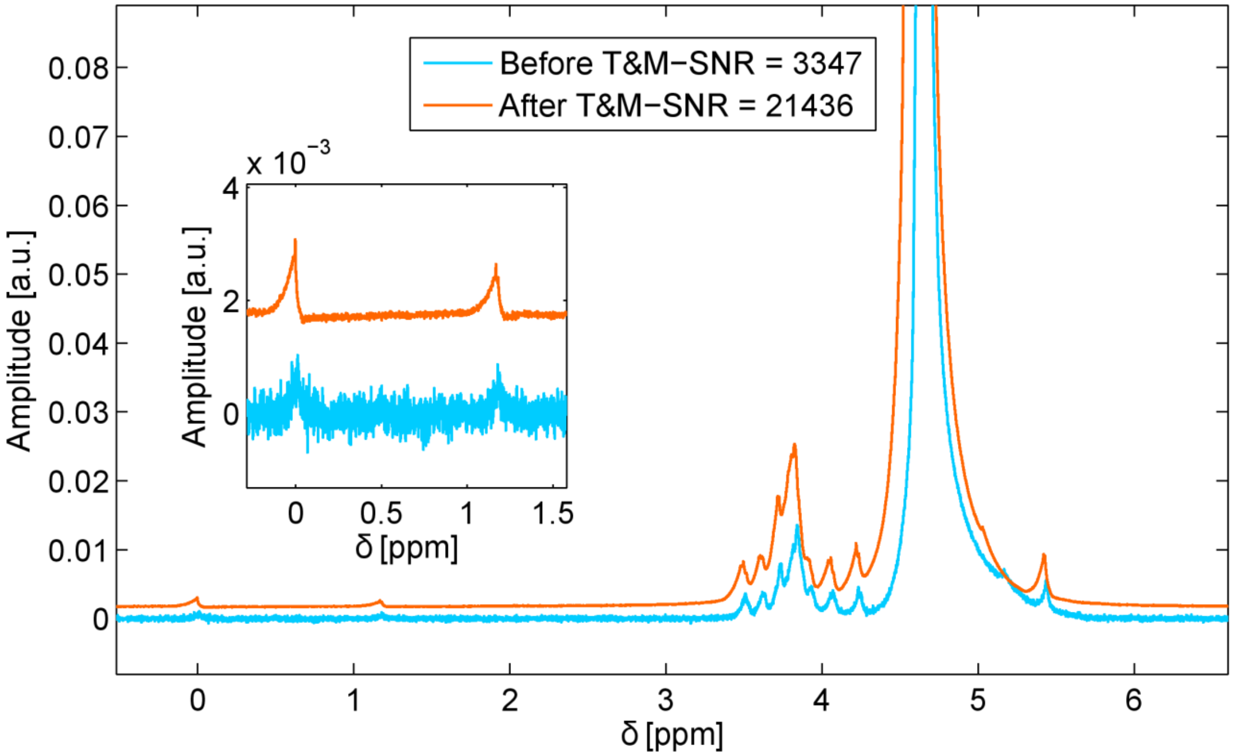

Fig. S2: Effect of tuning and matching on the quality of the NMR spectrum. The coil is a commercial 5 mm saddle coil designed to operate in a 11.7 T Bruker magnet at 500 MHz. The sample is 0.5 M NaPO3, 0.5 M Phosphoric Buffer Solution (PBS), 50 mM TSP, 0.5 M Sucrose. Readjusting the tuning and matching condition after loading the sample enhanced the SNR significantly (a factor of 6.4).

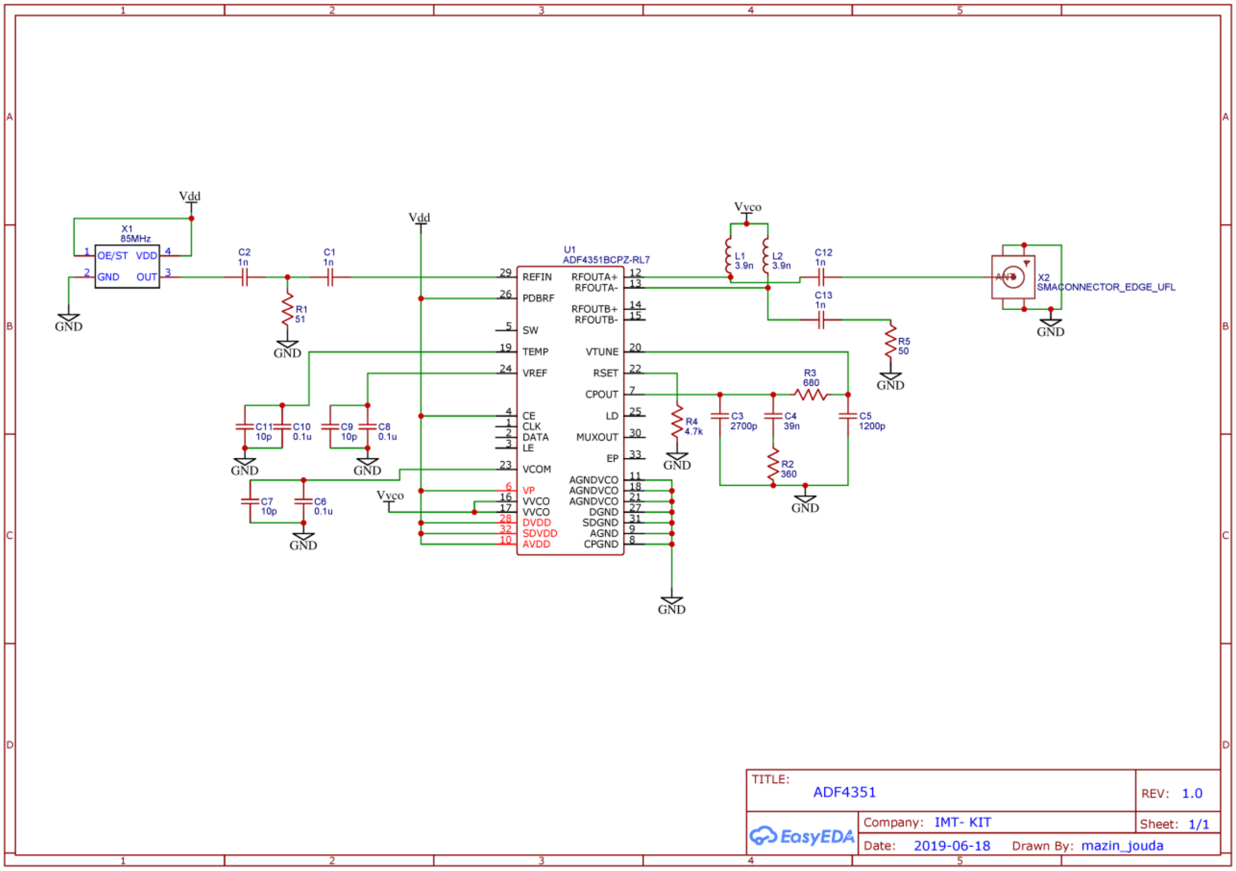

Fig. S3: Schematic diagram of the ADF4351 broadband frequency synthesizer.

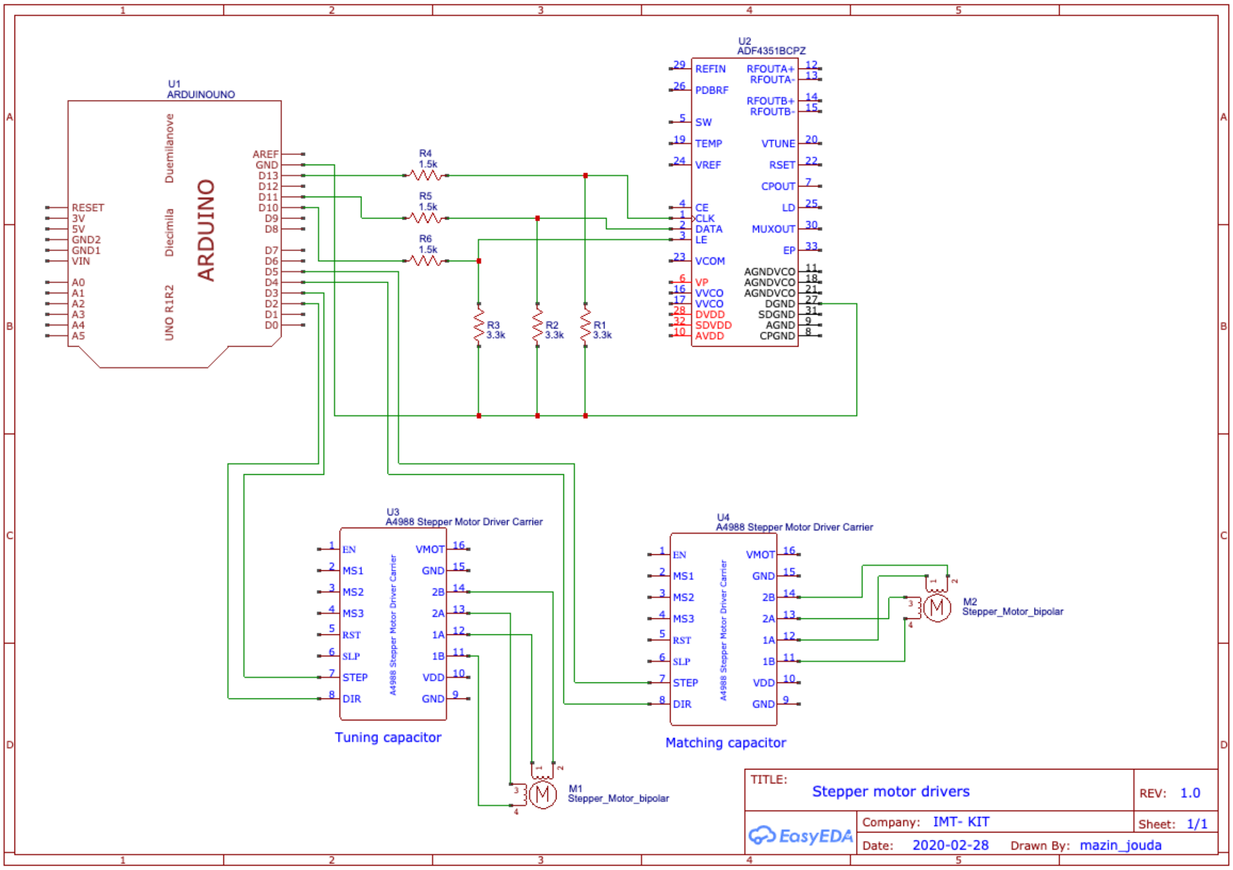

Fig. S4: Circuit diagram describing the interface between the Arduino and the ADF4351 frequency synthesizer. The diagram shows also the connections of the stepper motors' drivers (A4988).

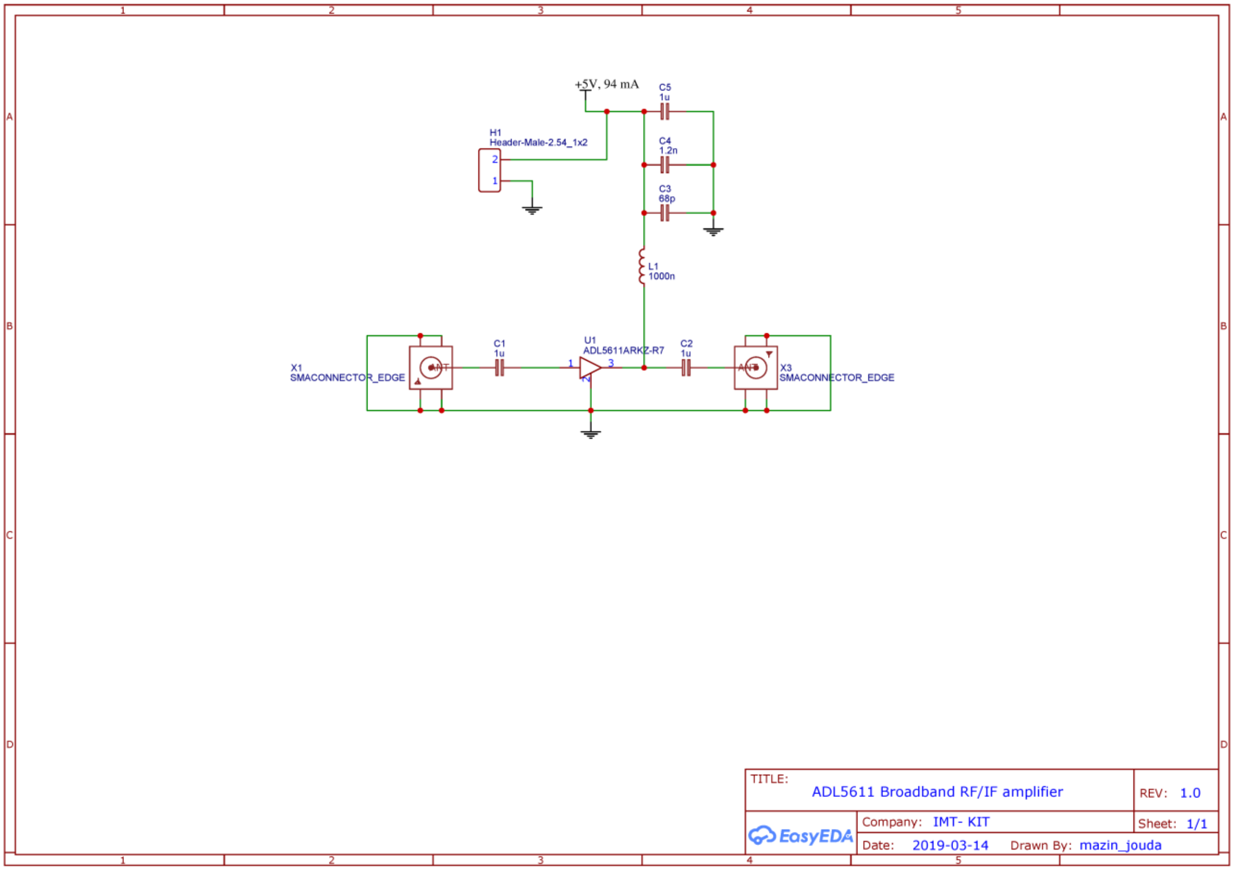

Fig. S5: Circuit diagram of the ADL5611 broadband IF/RF amplifier.

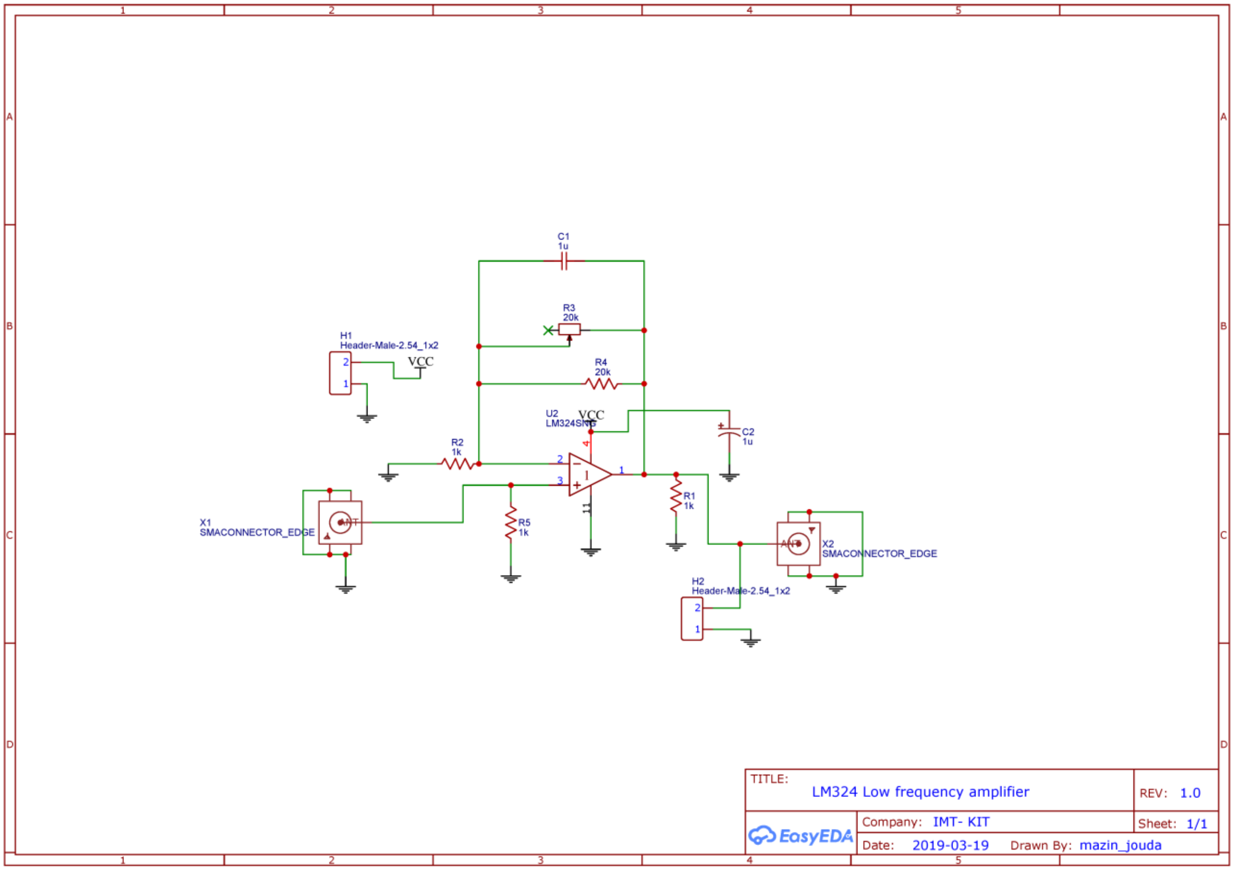

Fig. S6: Schematic of the LM324 low-frequency amplifier.
